# Cross-neutralizing activity of the chikungunya vaccine VLA1553 against three prevalent chikungunya lineages

**DOI:** 10.1080/22221751.2025.2469653

**Published:** 2025-02-25

**Authors:** Karin Kosulin, Trevor L. Brasel, Jeanon Smith, Maricela Torres, Annegret Bitzer, Katrin Dubischar, Vera Buerger, Robert Mader, Scott C. Weaver, David W.C. Beasley, Romana Hochreiter

**Affiliations:** a Valneva Austria GmbH, Vienna, Austria; b Department of Microbiology and Immunology, University of Texas Medical Branch, Galveston, TX, USA; c Institutional Office of Regulated Nonclinical Studies, University of Texas Medical Branch, Galveston, TX, USA; d Sealy Institute for Vaccine Sciences, University of Texas Medical Branch, Galveston, TX, USA; e Institute for Human Infections and Immunity, University of Texas Medical Branch, Galveston, TX, USA; f CRETA GmbH, Langeck im Burgenland, Austria

**Keywords:** Chikungunya virus, vaccine, VLA1553, cross-neutralization, CHIKV lineages

## Abstract

Cross-neutralization is generally a prerequisite for cross-protection of vaccines against diseases caused by heterologous viruses. Using sera obtained from a randomized clinical phase 3 trial in adults, we investigated the cross-neutralization activity of VLA1553, a vaccine recently approved to prevent chikungunya disease. Analysed in a plaque reduction neutralization test, the three major chikungunya virus (CHIKV) lineages, namely the East Central South African, the West African, and the Asian lineage, were inhibited by CHIKV-specific neutralizing antibodies present in the sera from vaccinated humans. This effect was independent of the time elapsed since vaccination. Moreover, the magnitude of the immune response was similar to the antibody levels detected in sera from convalescent chikungunya patients. Thus, VLA1553 has the potential to diminish the burden of chikungunya disease on a global scale.

Trial registration: ClinicalTrials.gov identifier: NCT04546724.

## Introduction

Viruses are known for their continuous evolution, a feature of their high mutation frequency and efficient adaptation in interaction with the host. The result of this process is the development of different lineages over time. Variability in the viral genomes provide a number of genotypes associated with different serological characteristics. Although this variability may leave immunization gaps when serotype specific vaccines are administered to protect from viral diseases (e.g. dengue or SARS-CoV-2), differences in the viral genome are not necessarily related to variable serological characteristics. In the case of CHIKV, strains are divided into three major lineages or genotypes but are present as a single serotype in infected patients [1,2]. A single serotype enables the development of a vaccine to protect against all circulating CHIKV lineages, East Central South African (ECSA), West African, and Asian [1–3].

CHIKV is one of the globally emerging viruses, now present in more than 100 countries [4]. CHIKV, a mosquito-borne arbovirus, is transmitted by *Aedes aegypti* and *Aedes albopictus* continuously extending endemic areas [5]. Associated with an increased risk of death for up to 84 days after symptom onset (cerebrovascular diseases, ischaemic heart diseases, diabetes) [6], the arthritogenic CHIKV causes a debilitating disease often associated with long-term sequelae such as persistent musculoskeletal symptoms or disabling polyarthritis severely hampering the quality of life of infected individuals [7,8]. Between 2019 and 2022, outbreaks in several countries led to high numbers of chikungunya infections such as in Brazil (656,000 cases), India (219,000 cases), Ethiopia (55,000 cases), Chad (41,000 cases), and Thailand (26,000 cases), indicating the global distribution of the disease [9]. The three major circulating CHIKV lineages have evolved and initially spread in different regions of the world. With an ECSA strain emerging in Kenya in 2004 and spreading to the Indian Ocean Island causing the La Réunion outbreak starting in 2005, a new ECSA sub-lineage was described (termed the Indian Ocean sub-lineage) [4,10]. Multiple point mutations in the virus’ envelope glycoproteins were associated with enhanced infection of the mosquito species *Aedes albopictus* [11-13].

In 2013, a strain from the Asian lineage emerged in the Caribbean followed in 2014 by the introduction of an ECSA strain in the north of Brazil, from where CHIKV expanded south infecting approximately 400,000 individuals in 2023 in the Americas [14–16].

The very recent U.S., Canadian, and European licensure of the live-attenuated vaccine VLA1553 (IXCHIQ^®^) (based on the La Reunion strain LR2006 OPY1 from the ECSA Indian Ocean sub-lineage) to prevent the disease caused by the CHIKV [17–20] raises the question of the vaccine’s coverage for the three globally circulating CHIKV lineages. The first results in non-human primate studies immunised with VLA1553 indicated cross-reactivity of the generated CHIKV-specific antibodies against the Caribbean strain (from the Asian lineage), which differs from the ECSA strain by 35 amino acids in the virus envelope glycoproteins [21]. To translate this effect into a clinical setting, sera of VLA1553-vaccinated individuals obtained from a phase 3 trial were used to analyse the neutralizing activity against the wild type CHIKV parental strain of VLA1553 derived from the ECSA, as well as against representative strains of the West African and Asian lineages.

## Material and methods

### Study design and samples

The sera analysed in this study were collected during a double-blind, multicentre, randomized, pivotal phase 3 trial enrolling healthy volunteers who received the chikungunya vaccine VLA1553 or placebo in the United States, i.e. in a population with expected minimal rate of pre-existing immunity against CHIKV (Code: VLA1553-301; ClinicalTrials.gov ID: NCT04546724) [22]. The trial was performed in compliance with the current International Council on Harmonisation/Good Clinical Practice guidelines and in accordance with the principles set forth in the Declaration of Helsinki (ethics approval: Advarra IRB; reference number Pro00045587). The clinical trial protocol specifically allowed for further testing of collected sera to assess cross-neutralization of heterologous CHIKV strains.

The primary endpoint of the phase 3 trial was the proportion of participants with a seroprotective CHIKV antibody level defined as 50% plaque reduction in a micro plaque reduction neutralization test (µPRNT) with a µPRNT_50_ titre ≥150 assessed 28 days post- vaccination in participants negative for CHIKV neutralizing antibodies at baseline [23]. For the µPRNT assay, a heterologous vaccinal strain from the Asian lineage (TSI-GSD-218) was used as previously described [22]. With a trial duration of 180 days, sera were collected at four different time points, i.e. at baseline, 28 days, 3 months and 6 months post-vaccination. In total, 72 samples from 32 trial participants were selected based on the results of a previous micro plaque reduction neutralization assay to include different timepoints as well as to cover the observed range of neutralization titres. This selection included ten samples at baseline (five seronegative and five seropositive participants from a total of 20 individuals with pre-existing neutralizing antibodies determined by µPRNT), 30 samples 28 days post-vaccination, 16 samples three months post-vaccination, and 16 samples collected six months post-vaccination.

### Viruses

In a qualified plaque reduction neutralization test (PRNT), the reactivity of the clinical trial sera was tested against wild type CHIKV strains of the three different lineages, specifically CHIKV ECSA isolated during the La Réunion outbreak in 2006 (the parental strain of the vaccine, LR2006_OPY1), the West African CHIKV lineage (strain 37997), and the Asian CHIKV lineage (strain Caribbean M109). Virus stocks were propagated in Vero cells (ATCC CCL-81) after rescue from plasmids containing the complete genome of the respective virus strains as described previously [12,24,25]. For this purpose, cells were electroporated (250 V, 10 ms pulse, three pulses with 1 sec interval) and subsequently cultivated for 48 h. The supernatant was collected, centrifuged at 8,000-10,000xg for up to 10 min, mixed with fresh growth medium and stored frozen ≤−65°C in aliquoted working stocks for each virus strain.

### Serologic assay

All work involving infectious CHIKV (or biological materials which may contain infectious virus) was performed in the Biosafety Level 3 laboratory at the University of Texas Medical Branch with personnel specifically trained in operational practices for work involving infectious or potentially infectious material.

Serum samples obtained during the clinical trial were shipped frozen on dry ice and stored at −80°C with temperature monitoring. Before analysis, samples were thawed at ambient temperature, heat inactivated (60 min at 56°C) and kept on ice. Positive control samples were aliquots of human serum samples collected after resolved natural infection with CHIKV (n = 3, Antibody Systems Inc.), whereas as negative control samples aliquots of CHIKV-negative human serum samples were used.

To perform the PRNT, Vero cells were seeded in 12-well plates at a density of 2×105 cells per well. On the following day, infectious CHIKV working stock diluted at concentration equivalent to 20–60 PFU/well was incubated with the same volume of serially two-fold diluted serum specimens at 37°C for one hour. This virus-serum mixture, with a starting serum dilution of 1:10 or 1:20, was then added to the pre-seeded Vero cells in triplicates. Following a one hour incubation at 37°C, 1 mL of overlay medium was dispensed in each well containing 1.25% (strains LR2006_OPY1 and 37997) or 2% (strain Caribbean M109) carboxymethylcellulose before incubation of plates at 37°C ± 2°C in a regulated incubator with 5% CO2 for 48 h ± 4 h (strains LR2006_OPY1 and Caribbean M109) or 36 h ± 4 h (strain 37997). For fixation of cells, 1–2 mL of 10% neutral buffered formalin was added to each well and allowed to incubate for a minimum of 30 min under ambient conditions. Following fixation, cells were washed to remove the neutral buffered formalin, then stained with 0.25% crystal violet solution for a minimum of 5 min. Stained plates were washed thoroughly with water and the assay acceptance was assessed with a standardized quality control procedure (expected plaque count of control wells, negative and positive control samples). Using plaque count data, the PRNT50 value was determined being as the highest dilution of serum that inhibits 50% of plaques in comparison to the average plaque count of the virus control. With a limit of detection for PRNT50 = 10, all results below 10 were imputed as PRNT50 = 5 in the data set.

### Statistical analysis

As an exploratory in vitro study, there was no underlying statistical hypothesis to be tested. CHIKV-specific neutralizing antibody titres are shown as geometric mean titre (GMT) with corresponding 95% confidence intervals (CI).

## Results

**Figure 1.**
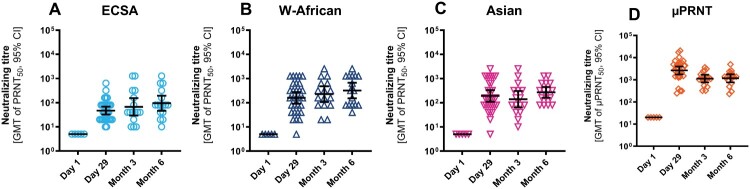
Cross-neutralization titres over time after vaccination with VLA1553. A, B, C: comparison of PRNT50 titres of the three investigated CHIKV lineages ECSA (strain LR2006_OPY1), West-African (strain WA_37997), and Asian (Caribbean_clone M109); D: corresponding µPRNT50 titres (strain: TSI-GSD-218) from the phase 3 clinical trial; only participants seronegative at baseline were included; data from vaccine sera are represented as GMT with 95% CI; limit of detection: PRNT50 = 10; all results below 10 were imputed as PRNT50 = 5 in the data set. limit of detection: µPRNT50 = 20.

The sera investigated in this study covered four collection time points including the primary endpoint of the clinical phase 3 trial set at 28 days post-vaccination. Post-vaccination sera from seronegative individuals demonstrated neutralizing antibody activity against the ECSA, the West African and the Asian lineages (Figure 1A, B, C). Assessed with the PRNT in 30 baseline seropositive and seronegative sera 28 days post-vaccination, the titres against the West African and the Asian lineage were not inferior to those of the ECSA lineage (ECSA: GMT = 47; 95% CI = 32-69; West African: GMT = 160; 95% CI = 92-279; Asian: GMT = 193; 95% CI = 111-334). For comparison, Figure 1D shows CHIKV-specific antibody titres of the same samples analysed with a neutralization assay applied in the phase 3 clinical trial [22]. This is a validated µPRNT assay, which follows a different protocol with a heterologous strain of the Asian lineage and resulted in generally higher values (GMT = 2647; 95% CI = 1,749–4,008).

The VLA1553 cross-neutralization activity over time recapitulated the observation from 28 days after vaccination with PRNT50 titres against the ECSA, the West African and the Asian lineage, throughout the trial period of 180 days (Figure 1A, B, C). The analysis of sera with the µPRNT assay again showed higher titre results (Figure 1D).

**Figure 2.**
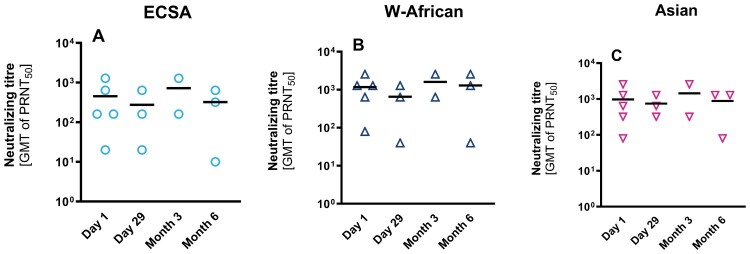
Absence of anamnestic response after vaccination with VLA1553. PRNT50 titres were analysed in samples from baseline positive participants with three CHIKV lineages: ECSA (strain LR2006_OPY1), West-African (strain WA_37997), and Asian (Caribbean_clone M109); data are represented as individual dots per sample and bars show the GMT; limit of detection: PRNT50 = 10.

In subjects seropositive at baseline, there was no anamnestic response observed as PRNT50 titres varied minimally during the trial period after vaccination with VLA1553 until day 180. Analogous to the outcomes in seronegative individuals, titres were similar across all tested CHIKV lineages (Figure 2A, B, C).

**Figure 3.**
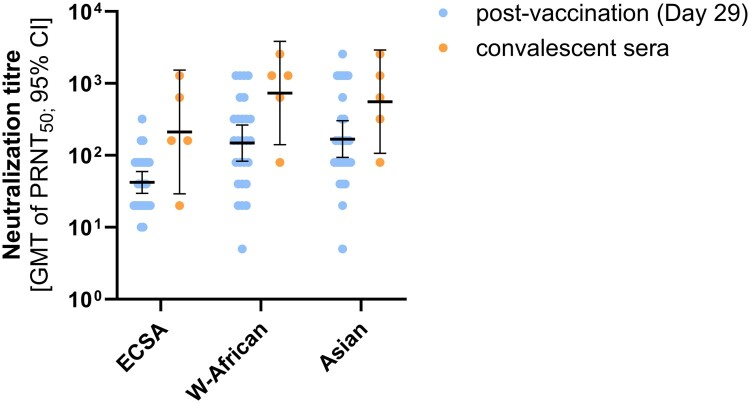
Comparison of PRNT50 titres from post-vaccination and convalescent sera. Neutralization titres from samples of baseline seropositive participants previously exposed to CHIKV (convalescent sera collected at day 1; n=5) and serum from individuals seronegative at baseline (post-vaccination sera collected at day 29; n=27) were analysed with three CHIKV lineages: ECSA (strain LR2006_OPY1), West-African (strain WA_37997), and Asian (Caribbean_clone M109). Data are represented as individual dots per sample and bars show the GMT with 95% CI; limit of detection: PRNT50 = 10; all results below 10 were imputed as PRNT50 = 5 in the data set.

To understand the magnitude of the generated immune response, the CHIKV-specific antibody titres from baseline positive samples collected at the day of vaccination (convalescent sera) are depicted together with titres from samples collected from baseline negative participants 28 days post-vaccination. Overlapping confidence intervals demonstrate that the immune response generated with VLA1553 matches the antibody levels from patients with a natural CHIKV infection in all three lineages (Figure 3).

## Discussion

The present study addresses neutralization coverage after CHIKV vaccination, a subject intensely researched in several vaccines against viral diseases.

Favoured by a single serotype of CHIKV, the development of a vaccine would meet the requirements to cover the currently circulating CHIKV lineages. After vaccination with VLA1553, our investigations showed a balanced coverage of neutralizing activity against strains representing the three main CHIKV lineages dominating on a global scale: the one from ECSA from which the vaccine VLA1553 originated, the one from the West African lineage, and the one from the Asian lineage. This *in vitro* cross-neutralization effect after immunization with VLA1553 was observed in baseline seronegative adults 28 days after immunization with lineage-specific variation in neutralization titres. However, this is not unexpected since initial studies of antigenic variation based on animals experimentally infected with two wild-type CHIKV strains spanning the known genetic diversity showed differences up to eight-fold in endpoint neutralization titres [26]. Homologous strain endpoint titres are usually but not consistently higher than homologous titres. For example, sera from macaques immunized with a Measles-vectored CHIKV vaccine, where the CHIKV genes were derived from an Indian Ocean lineage strain, sometimes had up to 16-fold higher endpoint titres against other CHIKV lineages, which is consistent with our results [27]. This inconsistency in homologous versus heterologous neutralizing antibody reactions may reflect differences in responses to B cell epitopes among outbred hosts.

Moreover, neutralization activity was independent of the time elapsed after vaccination with consistent results throughout the study period, i.e. sera collected up to six months after vaccination. Serum samples with seroprevalence at the day of vaccination, day 1 (neutralization activity referring to a previous natural CHIKV infection) showed slightly higher but comparable titres to samples collected at day 29 with seronegative baseline characteristics (see Figure 3) for all three CHIKV lineages tested. We are aware that only low numbers of samples (n = 5) from individuals with a natural CHIKV infection are included in this analysis. Also, the timepoint of the infection and the CHIKV lineage or strain are not known. However, the results are in concordance with data from a recent trial conducted in Brazil with our vaccine candidate VLA1553. In this trial we found comparable neutralizing titres for samples collected 28 days post-vaccination from baseline seronegative participants (n = 250) and samples obtained from seropositive participants (n = 52) indicating a similar magnitude of CHIKV-specific neutralizing antibody levels upon natural infection and vaccination [28].

In another recent study about cross-neutralizing activity of VLA1553, sera from subjects immunized with VLA1553 were investigated to assess their cross-neutralizing activity against other arthritogenic alphaviruses such as o’nyong-nyong virus, Ross River virus, and Mayaro virus. Using a PRNT similar to the test used in this study, this investigation showed broad activity against all three alphaviruses as well as against several other CHIKV strains with activity persisting over the whole observation period of one year [29]. Considering the global situation where endemicity of CHIKV is expanding, the role of antibodies induced by vaccination to neutralize strains from all lineages will become increasingly important in the near future.

We currently see CHIKV transmission by the vectors Ae. aegypti, and increasingly in some locations by Ae. albopictus, a more zoophilic mosquito species [30], spreading the virus to more temperate regions such as Europe with infections reported in Italy, Spain and France [31-33]. Expansion of CHIKV northward to countries such as Germany or the Benelux states seems not to be excluded in the near future [34]. In parallel, the cold-tolerant vector Ae. geniculatus has been found to be highly susceptible to CHIKV infection possibly adding an indigenous European mosquito species as potential vector for transmission [35]. Taken together, these data strongly emphasize the benefit of a vaccine with broad coverage able to prevent the disease caused by CHIKV independent of the lineage.

In the clinical trials investigating the immune response of VLA1553, a µPRNT assay was used yielding at least four times higher analysis results than the PRNT50 assays used here. The assays, µPRNT and PRNT, differ in some essential features of their protocol. The µPRNT uses immunostaining and automated scanning for detection of plaques, which is much more sensitive than manual counting in the PRNT. In addition, variations in cell stocks and incubation times have to be considered. Thus, a difference in the numerical read-outs was expected. For the µPRNT assay, a clinical surrogate of protection was accepted by the regulatory bodies based on human transfer studies in non-human primates. This value has been defined as µPRNT50 ≥150 using a conservative approach [23]. Despite a decrease of CHIKV specific antibody titers after day 29, this defined seroprotection value for the µPRNT assay was also well exceeded with a GMT of 784.7 at 2 years post-vaccination [36]. Although no direct comparison of measured titre levels can be made between different assay formats and individual CHIKV strains, the detection of neutralizing antibodies in post-vaccination sera supports the understanding that most antibodies generated against a single strain recognize CHIKV of different lineages.

Cross-protection may also result from previous exposure to the CHIKV as observed in Cambodia, emphasizing the lasting immune response induced by a single serotype of the CHIKV [1,37]. Thus, these first data on cross-neutralization of VLA1553 of the three global CHIKV lineages suggest its cross-protective effect. Similar to our results, in a first-in-human trial of a candidate adenovirus vector CHIKV vaccine, complete seroconversion was achieved with broad cross-neutralizing activity with a CHIKV-specific titre variation up to five-fold against the three main CHIKV lineages investigated in this study and also the Indian Ocean lineage evolved from the ECSA lineage [38].

Heterologous cross-neutralizing activity has been recently described for another CHIKV vaccine [39]. In that study, antibodies generated by a virus-like particle vaccine against CHIKV were cross-neutralizing to the closely related o′nyong-nyong virus and, to a lesser degree, Mayaro virus. Similar observations were made in mice after infection with one alphavirus followed by analysis of the cross-reactivity upon challenge with another alphavirus confirming cross-neutralization between CHIKV and o’nyong-nyong virus [40], which has been confirmed in humans with cross-neutralizing antibodies against other arthritogenic alphaviruses after CHIKV infection [41].

As shown in a previous trial of VLA1553, there was no anamnestic response in individuals with pre-existing antibodies against CHIKV [42]. This is likely due to the pre-existing immunity blocking the uptake of the vaccine in the recipient, and not a limiting feature of the vaccine as data from Yoon and co-workers indicate that every measurable anti-CHIKV titre may be considered as protective [43].

The recent US, European and Canadian licensure of VLA1553 under the brand name IXCHIQ® for the prevention of the disease caused by CHIKV is indicated for adults who are at increased risk of exposure to the virus [17]. Of note, there is no age limit for older people as they all achieved protective CHIKV-neutralizing titres [22].

This study has several limitations. With a total of 72 sera from the pivotal phase 3 trial, only the timepoint representing the primary endpoint of the VLA1553-301 pivotal trial set at 28 days after vaccination was covered by 30 samples, whereas all sera collected at other trial periods were less represented. Due to the limited number of samples, selection was based on titre levels known from the clinical trial testing (µPRNT) rather than the follow-up of individual trial participants to describe titre persistence over time. The use of functionally similar but different assays, i.e. PRNT and µPRNT, in the present in vitro study and the clinical trial, aggravated the interpretation of data as the correlation between the assays is not linear. Finally, this comparison was performed with an in vitro assay and confirms what is known from cohort studies, but needs affirmation in clinical trials with efficacy endpoints. Despite these shortcomings and the need for extended investigations based on statistical sample size calculations, this study provides valuable insights into the nature of the immune response triggered by vaccination with VLA1553.

In conclusion, in vitro testing of three different and representative CHIKV lineages suggests that VLA1553 confers robust cross-neutralization. This effect is independent of the time elapsed since vaccination with persisting titres over time. The magnitude of the humoral immune response following vaccination seems to reach antibody levels comparable to those detected in sera from convalescent chikungunya patients who are believed to possess life-long protection from the disease caused by the CHIKV.

## Author contributions

K.K., K.D. and R.H. conceived and designed the project. J.S., M.T., and T.B. performed neutralization experiments; J.S. and M.T. assisted with data handling/analysis; D.B. did data analysis and report writing for the VLA1553-301 testing at UTMB; D.B. and S.W. contributed as subject matter experts; T.B. was primarily responsible for data organization and consolidation. K.K., R.H., and R.M. wrote the manuscript. A.B., K.D., V.B., K.K., R.H., and R.M. analysed the data for manuscript writing. All of the authors approved the final manuscript.

## Supplementary Material

supplementary Table.docx
