# Supplementary material for: Cross-neutralizing activity of the chikungunya vaccine VLA1553 against three prevalent chikungunya lineages
Source: Emerg Microbes Infect. 2025 Feb 25;14(1):2469653. doi: 10.1080/22221751.2025.2469653 (PMC11894744; doi:10.1080/22221751.2025.2469653)
Supplement: supplementary Table.docx [file TEMI_A_2469653_SM8733.docx]

**Supplementary Table**

| **Sample Information** | | **ECSA (LR 2006 OPY1)** | **West African (37997)** | **Asian (Caribbean M109)** |
| --- | --- | --- | --- | --- |
| **Subject ID** | **Visit** | **PRNT50 dilution endpoint** | **PRNT50 dilution endpoint** | **PRNT50 dilution endpoint** |
| 1553-1-04-016 | Visit 1 - Day 1 | 5 | 5 | 5 |
| 1553-1-04-016 | Visit 3 - Day 29 | 20 | 160 | 80 |
| 1553-1-04-016 | Visit 4 - Day 85 | 40 | 320 | 80 |
| 1553-1-04-017 | Visit 1 - Day 1 | 5 | 5 | 5 |
| 1553-1-04-017 | Visit 3 - Day 29 | 20 | 320 | 20 |
| 1553-1-04-017 | Visit 4 - Day 85 | 10 | 640 | 40 |
| 1553-1-04-023 | Visit 1 - Day 1 | 5 | 5 | 5 |
| 1553-1-04-023 | Visit 3 - Day 29 | 20 | 1280 | 1280 |
| 1553-1-04-023 | Visit 5 - Day 180 | 80 | 1280 | 320 |
| 1553-1-04-025 | Visit 1 - Day 1 | 5 | 5 | 5 |
| 1553-1-04-025 | Visit 3 - Day 29 | 20 | 640 | 80 |
| 1553-1-04-025 | Visit 5 - Day 180 | 80 | 1280 | 320 |
| 1553-1-04-030 | Visit 3 - Day 29 | 20 | 160 | 40 |
| 1553-1-04-030 | Visit 5 - Day 180 | 40 | 1280 | 160 |
| 1553-1-04-033 | Visit 3 - Day 29 | 40 | 320 | 160 |
| 1553-1-04-033 | Visit 5 - Day 180 | 160 | 1280 | 640 |
| 1553-1-04-037 | Visit 3 - Day 29 | 20 | 1280 | 160 |
| 1553-1-04-037 | Visit 5 - Day 180 | 80 | 320 | 320 |
| 1553-1-04-038 | Visit 3 - Day 29 | 40 | 1280 | 160 |
| 1553-1-04-038 | Visit 4 - Day 85 | 1280 | 320 | 160 |
| 1553-1-04-040 | Visit 1 - Day 1 | 5 | 5 | 5 |
| 1553-1-04-040 | Visit 3 - Day 29 | 80 | 80 | 320 |
| 1553-1-04-040 | Visit 4 - Day 85 | 40 | 1280 | 640 |
| 1553-1-04-041 | Visit 3 - Day 29 | 80 | 640 | 1280 |
| 1553-1-04-041 | Visit 4 - Day 85 | 20 | 160 | 80 |
| 1553-1-04-043 | Visit 3 - Day 29 | 40 | 320 | 40 |
| 1553-1-04-043 | Visit 5 - Day 180 | 1280 | 80 | 80 |
| 1553-1-04-087 | Visit 3 - Day 29 | 20 | 5 | 5 |
| 1553-1-04-087 | Visit 4 - Day 85 | 10 | 80 | 10 |
| 1553-1-06-001 | Visit 3 - Day 29 | 160 | 320 | 80 |
| 1553-1-06-001 | Visit 4 - Day 85 | 40 | 320 | 80 |
| 1553-1-06-007 | Visit 3 - Day 29 | 80 | 1280 | 1280 |
| 1553-1-06-007 | Visit 4 - Day 85 | 40 | 1280 | 320 |
| 1553-1-06-023 | Visit 3 - Day 29 | 80 | 160 | 2560 |
| 1553-1-06-023 | Visit 5 - Day 180 | 80 | 320 | 640 |
| **Sample Information** | | **ECSA (LR 2006 OPY1)** | **West African (37997)** | **Asian (Caribbean M109)** |
| **Subject ID** | **Visit** | **PRNT50 dilution endpoint** | **PRNT50 dilution endpoint** | **PRNT50 dilution endpoint** |
| 1553-1-06-024 | Visit 3 - Day 29 | 80 | 80 | 1280 |
| 1553-1-06-024 | Visit 4 - Day 85 | 320 | 80 | 180 |
| 1553-1-06-042 | Visit 3 - Day 29 | 320 | 40 | 320 |
| 1553-1-06-042 | Visit 5 - Day 180 | 640 | 640 | 320 |
| 1553-1-06-048 | Visit 3 - Day 29 | 80 | 320 | 1280 |
| 1553-1-06-048 | Visit 4 - Day 85 | 80 | 160 | 40 |
| 1553-1-08-002 | Visit 3 - Day 29 | 80 | 80 | 80 |
| 1553-1-08-002 | Visit 5 - Day 180 | 20 | 80 | 80 |
| 1553-1-08-005 | Visit 3 - Day 29 | 80 | 160 | 160 |
| 1553-1-08-005 | Visit 5 - Day 180 | 40 | 160 | 160 |
| 1553-1-08-006 | Visit 3 - Day 29 | 40 | 160 | 160 |
| 1553-1-08-006 | Visit 5 - Day 180 | 80 | 80 | 640 |
| 1553-1-08-022 | Visit 1 - Day 1 | 640 | 1280 | 1280 |
| 1553-1-08-022 | Visit 3 - Day 29 | 640 | 640 | 640 |
| 1553-1-08-022 | Visit 5 - Day 180 | 640 | 1280 | 1280 |
| 1553-1-08-027 | Visit 3 - Day 29 | 20 | 20 | 80 |
| 1553-1-08-027 | Visit 4 - Day 85 | 10 | 20 | 20 |
| 1553-1-08-073 | Visit 3 - Day 29 | 10 | 20 | 80 |
| 1553-1-08-073 | Visit 4 - Day 85 | 40 | 20 | 80 |
| 1553-1-11-005 | Visit 3 - Day 29 | 40 | 40 | 640 |
| 1553-1-11-005 | Visit 4 - Day 85 | 40 | 80 | 320 |
| 1553-1-11-052 | Visit 3 - Day 29 | 20 | 40 | 80 |
| 1553-1-11-052 | Visit 5 - Day 180 | 80 | 80 | 160 |
| 1553-1-11-134 | Visit 3 - Day 29 | 160 | 80 | 160 |
| 1553-1-11-134 | Visit 4 - Day 85 | 320 | 160 | 1280 |
| 1553-1-17-008 | Visit 3 - Day 29 | 10 | 20 | 40 |
| 1553-1-17-008 | Visit 5 - Day 180 | 20 | 80 | 80 |
| 1553-1-23-020 | Visit 1 - Day 1 | 20 | 80 | 80 |
| 1553-1-23-020 | Visit 3 - Day 29 | 20 | 40 | 320 |
| 1553-1-23-020 | Visit 5 - Day 180 | 10 | 40 | 80 |
| 1553-1-32-161 | Visit 1 - Day 1 | 160 | 640 | 320 |
| 1553-1-32-161 | Visit 4 - Day 85 | 160 | 640 | 320 |
| 1553-1-32-165 | Visit 1 - Day 1 | 1280 | 2560 | 2560 |
| 1553-1-32-165 | Visit 4 - Day 85 | 1280 | 2560 | 2560 |
| 1553-1-41-013 | Visit 1 - Day 1 | 160 | 1280 | 640 |
| 1553-1-41-013 | Visit 3 - Day 29 | 160 | 1280 | 1280 |
| 1553-1-41-013 | Visit 5 - Day 180 | 320 | 2560 | 1280 |

NOTE: The lowest and highest dilutions tested were 1:10 and 1:2,560, respectively;

Titer values <10 are imputed with 5; titer values >2,560 were reported as 2,560
